# Ambient air pollution and respiratory mortality in Xi’an, China: a time-series analysis

**DOI:** 10.1186/s12931-019-1117-8

**Published:** 2019-07-05

**Authors:** Kingsley Katleho Mokoena, Crystal Jane Ethan, Yan Yu, Karabo Shale, Feng Liu

**Affiliations:** 1 School of Public Health, Health Science Center, Xi’an Jiaotong University, 76, Yanta West Road, Xi’an, 710061 Shaanxi Province China; 2 Department of Life Sciences, Central University of Technology, Free State, Bloemfontein, Free State 9300 South Africa; 3 Department of Environmental and Occupational Studies, Cape Peninsula University of Technology, Cape Town, 8000 South Africa; 4 Shaanxi Provincial Center for Disease Control and Prevention, Xi’an, 710054 Shaanxi China

**Keywords:** Air pollution, Environmental exposure, Principal component analysis, Public health, Respiration disorders

## Abstract

**Background:**

Although air pollution is a known fundamental problem in China, few studies have investigated the associations between ambient air pollution and respiratory mortality in non-metropolitan cities of China. The study aimed to investigate a potential relationship between short-term exposure to ambient air pollutants and respiratory mortality in Xi’an, China.

**Methods:**

Daily averages of PM_2.5_, SO_2_, O_3_, temperature, relative humidity and daily counts of respiratory mortality were obtained (2014–2016). Using a single and multi-pollutant approach in time-series analysis, the generalized additive model with natural splines was used for analysis. Subgroup analysis stratified by gender and age group (≤ 64 years and ≥ 65 years) was conducted.

**Results:**

Seven thousand nine hundred sixty-five cases of respiratory mortality were assessed, with 62.9, 28.5, and 8.6% of mortality attributed to chronic lower respiratory diseases, influenza and pneumonia, as well as other forms of respiratory diseases, respectively. Observed pollutants were significantly associated with respiratory mortality. In the single pollutant model, 10 μg/m^3^ increase in a two-day moving average of PM_2.5_, and SO_2_ concentrations were significantly associated with relative risk 1.313(1.032, 1.708) and 1.4020(0.827, 2.854) of respiratory mortality, respectively. The effects of both air pollutants remained statistically significant after adjusting for collinearity in the multi-pollutant model. Ozone was only statistically associated with respiratory mortality in females at lag 0 [RR: 0.964(0.938, 0.991)].

**Conclusion:**

This study provided evidence that respiratory mortality in Xi’an was significantly associated with exposure to ambient air pollutants from 2014 to 2016.

## Highlights

- Chronic lower respiratory diseases lead respiratory mortality in Xi’an.
- From all air pollutants, PM_2.5_ revealed stronger effects on respiratory mortality.
- O_3_ was statistically associated with risk of respiratory mortality in females (lag 0).

## Introduction

Urbanization and industrialization have been major contributing factors to the ongoing change in global climate, with increased air pollution and poor air quality being some of the main consequences thereof. As the global climate and air quality deteriorates, exposure to air pollution remains a fundamental concern to public health.

In recent years, developing countries in Asia like India and China have embarked on major urbanization and industrialization and consequently have been struggling immensely with severe air pollution. Various studies and health-related agencies have presented evidence that reveals ambient air pollution continues to increase daily particularly in such developing countries, resulting in various health effects and fatalities on the general population. In 2008 approximately 1.3 million deaths were attributed to ambient (outdoor) air pollution exposure, and tripled to approximately 3.7 million deaths in 2012; directly stating that more than 50% of air pollution-related deaths (7 million) globally was due to outdoor air pollution only [1, 2]. The World Health Organization also estimated that in 2016 ambient air pollution was responsible for 4.2 million premature deaths globally [3].

Outdoor air pollution in China is undoubtedly a severe environmental and health concern; however, explicit details about ambient air pollution patterns in China, its similarities or differences from other countries and associated adverse health effects in rural areas and small cities are unknown. Some studies have reported the relationship between various air pollutants and health effects, with respiratory diseases (morbidity and mortality) remaining on the forefront in many Chinese cities and globally [4–7]. The respiratory system is directly and easily exposed to the external environment, making it more susceptible to the effects and influences of the surrounding environment. Subsequently, air pollution has been widely acknowledged as a major influence and exacerbating factor on various respiratory diseases such as lung cancer, bronchitis, chronic obstructive pulmonary diseases, pneumonia, asthma, and influenza. Globally, respiratory diseases remain in the top three leading causes of non-communicable diseases (NCD’s) mortality, and therefore it is highly essential to identify related risk factors and appropriate preventive measures clearly. In China, chronic respiratory diseases remained in the top five leading causes of mortality in 2016, accounting for 9% (approximately 870,000 deaths) of the total NCD deaths (9259,000) [8]. Despite the continuous effort and significant improvements in ambient air quality in China over the past decade, NCD’s were responsible for approximately 89% of all total mortality in 2016, a figure slightly higher than the global proportion of 60% for NCD’s [8, 9]. With respiratory diseases continuing to be on the rise, the World Health Organization predicts by 2030 respiratory diseases will be the leading cause of morbidity and mortality [10].

Although strong epidemiological evidence linking exposure to ambient air pollutants and respiratory mortality exists in China (particularly in the metropolis), an urgent need for experimental studies to adequately describe the associations and mechanisms that triggers this health outcome in non-metropolis cities like Xi’an remains. With short-term exposure to ambient air pollution having a significant public health impact that can no longer be neglected, as well as the fact that the complex nature of ambient air pollutants and health effects varies by location; the objective of this study was to assess a potential association between exposure to ambient air pollutants and respiratory mortality in Xi’an.

## Materials and methods

### Study area and population

The study area included 13 districts of Xi’an (34°15′44″N, 108°56′16″E) with the population being residents in the Household Register of Xi’an, China. With an area of 9,983 km^2^ and a population of 8,705,600 million inhabitants, Xi’an is the largest city in Central North-Western China. It is located in the middle of the Yellow River’s Guangzhong Plain, with Qinling Mountains to the south and Weihe River to the north. Xi’an has a distinct climate pattern of four seasons per year, corresponding to the sub-humid and temperate continental monsoon climate. As an important industrial epicenter, Xi’an carries the burden of poor air quality in the region experiencing severe air pollution problem [11].

### Study design

A time-series study design was used for the current study to assess the respiratory mortality risks associated with short-term exposures to ambient air pollution (PM_2.5_, SO_2_, and O_3_) and meteorological parameters (temperature (°C) and relative humidity (%)) from 2014 to 2016 using daily aggregate data.

### Data collection

Daily respiratory mortality records from January 1, 2014, to June 2, 2016, were validated and acquired from the Shaanxi Centre for Disease Control and Prevention in Xi’an. Patient’s information on the death records included nationality, gender, age, diagnosis, date of death, and the cause of death recorded according to the *International Classification of Diseases, Tenth Revision* (ICD-10), chapter X: respiratory mortality (J00–99) and further grouped according sex (male and female) and age (≤ 64 years and ≥ 65 years) [12, 13]. Daily average records of ambient air pollutants (μg/m^3^) were collected from the Xi’an Environmental Monitoring Centre (generated from the 13 state-controlled monitoring stations across Xi’an) over the same duration. Meteorological data was derived from the China Meteorological Data Sharing Service System over the same period.

### Statistical methods

To explore the association between ambient air pollutants and respiratory mortality, a generalized additive model with natural splines were constructed to link data by date and analyze the associations of daily respiratory mortality, daily concentrations of ambient air pollutants (PM_2.5_, SO_2_, and O_3_), and other related covariates [14, 15]. A basic model was constructed without the inclusion of the air pollutants. To adjust for confounding effects, natural splines functions of time and meteorological parameters were incorporated into the model, while the day of the week was included as a dummy variable [16]. Sensitivity analysis was conducted using various degrees of freedom (*df*) per year (4 *df*/year, 8 *df*/year and 12 *df*/year) for the time trend and 3 *df* was used for temperature and relative humidity [17, 18]. After examining the effects of variables in the basic model, the air pollutants were incorporated into the model to note differing changes on respiratory mortality. The model used to obtain the relative risk associated with respiratory mortality due to air pollution exposure is given as follows:

$$ Log\left[E\left({Y}_t\right)\right]={\propto}_0+\sum \limits_{i=1}^q{\beta}_i\left({X}_i\right)+\sum \limits_{j=i}^p{f}_j\left({Z}_j, df\right)+{W}_t(week) $$

Where: *E(Y*_*t*_*)* indicates the number of expected at day *t*, β indicates the log-relative rate of respiratory mortality related to a unit increase of air pollutants, *X*_*i*_ represents the concentration of air pollutants at day *t,* with *W*_*t*_*(week)* being the dummy variable for day of the week, *Z*_*j*_ represents the predictor variables (i.e., time, mean daily temperature and relative humidity) and *fj* indicates smooth function of these predictor variables. The effects of environmental stressors (such as air pollutants) are sometimes not observed instantly but are delayed. Therefore, to observe the aspect of time in revealing some delayed effects of the air pollutants on respiratory mortality, a lag analysis was conducted using the following lag structures [single-day lag (0–4) and multi-day lag (01–04)]. For the lag analysis, a single day concentration of air pollution (lag 0-current day only) and a 2-day average of air pollutant concentration (lag 01-current-day and previous-day) were used to determine the single day and cumulative effects of the pollutants [19, 20].

With the single pollutant model, it is easy to assess the interaction between the predictor (air pollution) and response (respiratory mortality) variables. However, air pollutants in the atmosphere are not present in individual forms and possibly interact with each other, suggesting they may have a combined effect on a health outcome. To assess and understand this possible combined effect, a multi-pollutant model was formulated, to analyze three major air pollutants simultaneously. In a multivariate regression analysis such as this, a situation may occur where the predictor variables are highly correlated with each other as well as with the response variable, and this is referred to as “collinearity”. A principal component was hence introduced into the multi-pollutant model to establish an exposure-response outcome without the impacts of collinearity between the air pollutants [21]. Relative risks and their confidence intervals (CIs) were estimated to quantify the influence of each air pollutant on respiratory mortality. Analyses were stratified by gender (male and female) and age group (≤ 64 years and ≥ 65 years) to examine potential effect per subgroup. All results were presented as relative risk (95%CI) of daily respiratory mortality associated with every 10 μg/m^3^ increase in ambient air pollutant concentration. Statistical analyses were conducted using R software version 3.2.2. The “mgcv” package was used to fit the generalized additive model. The statistical significance in all analyses was defined as *P* < 0.05.

## Results

### Descriptive statistics

Descriptive statistics for daily respiratory mortality counts, ambient air pollutants concentration and meteorological parameters are presented in Table 1. A total of 7,965 cases of respiratory mortality occurred in Xi’an during the study. Approximately 62.9% of the mortality was attributed to chronic lower respiratory diseases (ICD-10: J40–47), 28.5% to influenza and pneumonia (ICD-10: J09–18) and 8.6% to other forms of respiratory diseases. Based on the city’s population, the respiratory mortality rate in Xi’an was estimated as 31 per 100,000 people with a proportional mortality rate of 6 per 1,000 people during the same period. Based on the GB3095–2012, PM_2.5_ average concentration was higher than the annual limit for PM_2.5_ in China; all other pollutants were below their respective annual limits [22].

**Table 1:** Descriptive statistics of daily mortality counts, air pollutant concentrations, and meteorological parameters

	Mean ± SD	Min	P25	P50	P75	Max
Respiratory diseases	9 ± 3.95	1	6	9	11	23
Male	5.63 ± 2.84	1	3	5	7	17
Female	3.59 ± 1.98	1	2	3	5	11
≤ 64 years	1.90 ± 1.07	1	1	2	2	8
≥ 65 years	3.22 ± 1.81	1	2	3	4	10
Air pollutant concentration (μg/m^3^)^a^						
PM_2.5_	66.63 ± 50.13	11	36	53	77.25	527
SO_2_	26.38 ± 21.55	2	12	19	35	145
O_3_	39.62 ± 24.45	5	20	33	56	117
Meteorological parameters						
Temperature (°C)	13.48 ± 9.60	−8	5	14	22	34
Humidity (%)	65.43 ± 15.8	21	55	65	78	99

^*a*^ *24-hour averages for PM*_*2.5*_ *, and SO*_*2*_ *; the maximal 8-h average for O*_*3*_

*SD: standard deviation; Px (xth percentiles)*

### Correlation analysis

Table 2 shows the correlation between ambient air pollutants and meteorological parameters. Spearman’s correlation was used. PM_2.5_ and SO_2_ were positively correlated. Ozone on the other had a negative correlation with both PM_2.5_ and SO_2_ but presented a significant positive correlation with temperature. However, other pollutants were negatively correlated with temperature. Humidity was also negatively correlated with all air pollutants except PM_2.5_ in addition to temperature. Correlation of the above indexes displayed possible collinearity of the various independent variables.

**Table 2:** Spearman correlation coefficients between daily ambient air pollutant and meteorological parameters

	PM_2.5_	SO_2_	O_3_	Temperature
SO_2_	0.670**			
O_3_	− 0.441**	− 0.666**		
Temperature	−0.431**	−0.786**	0.778**	
Humidity	0.041	−0.248**	−0.240**	0.058

**P < 0.01

### Graphical representation of mortality and air pollutants data

**Fig. 1:**
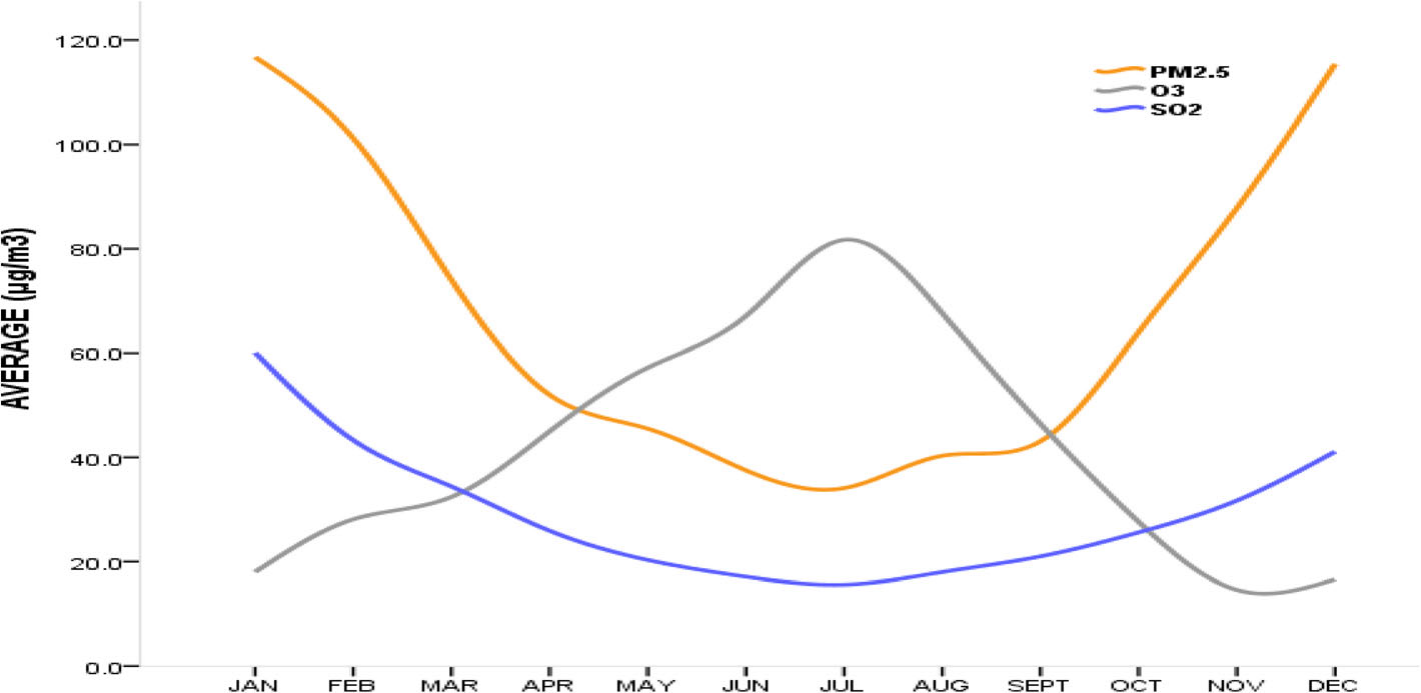
Average concentration levels for ambient air pollutants in Xi’an from 2014 to 2016

The climate of Xi’an is characterized by four seasons, with each season displaying different levels of air pollutants concentration. Figure 1 shows PM_2.5,_ and SO_2_ concentration levels were high during the cold seasons [Autumn (Sept-Nov) and Winter (Dec-Feb)] and low during the warm seasons [Spring (Mar-May) and Summer (Jun-Aug)]; however, this was reversed for O_3_. For all seasons, SO_2_ and O_3_ were below the daily ambient air quality limits of 150 μg/m^3^ and 160 μg/m^3^ respectively. PM_2.5,_ on the other hand, was below the daily ambient air quality limits of 75 μg/m^3^ for all seasons except winter [22].

**Fig. 2:**
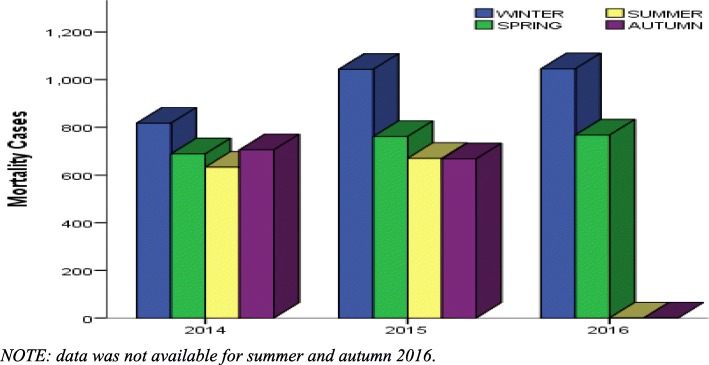
Seasonal distribution of respiratory morality in Xi’an from 2014 to 2016

Seasonal distributions of respiratory mortality are illustrated in Fig. 2. Respiratory mortality was highest in winter (all years) and lowest in summer for 2014 and 2015.

### Statistical analysis results

Statistical analysis carried out to determine the effects of ambient air pollution on respiratory mortality showed that increase in every 10 μg/m^3^ of PM_2.5_ and SO_2_ concentrations were significantly associated with risk of respiratory mortality (Table 3). After adjusting for collinearity by principal component analysis in the multi-pollutant model, there was a slight increase in the risk estimates which was also statistically significant. For O_3_, no significant association was noted for the risk of respiratory mortality from the single pollutant and multi-pollutant analysis.

**Table 3:** Relative risk of daily respiratory mortality associated with 10 μg/m^3^ increase in air pollutant concentration

	RR(95% CI)	*P-*value
Single model		
PM_2.5_	1.313 (1.032, 1.708)	0.024
O_3_	1.833 (0.604, 8.633)	0.437
SO_2_	1.4020 (0.827, 2.854)	0.042
After adjusting for collinearity by principal component analysis		
PM_2.5_	1.328 (1.033, 1.745)	0.019
O_3_	2.061 (0.630, 10.198)	0.746
SO_2_	1.524 (0.836, 3.406)	0.015

### Lag effects

Using single-day (lag 0–4) and cumulative (lag 01–04) lags, the relative risk associated with respiratory mortality due to lag effects (delayed effects) of air pollutants concentration was analyzed. As shown in Table 4, PM_2.5_ in the single pollutant model was significantly associated with the risk of respiratory mortality at lag 2 and lag 02; and in the multi-pollutant model at lag 0, lag 2, and lag 02. Statistically significant associations were also observed for SO_2_ in the single pollutant model at lag 4 and in the multi-pollutant model at lag 4 and lag 02.

**Table 4:** RR for significant associations between air pollutants and daily respiratory mortality due to lag effects

	Lag	PM_2.5_	O_3_	SO_2_
Single Model	Lag 0	1.001 (0.996, 1.005)	0.995 (0.979, 1.010)	0.995 (0.979, 1.011)
	Lag 1	1.002 (0.997, 1.006)	1.001 (0.984, 1.017)	0.991 (0.977, 1.004)
	Lag 2	1.003 (1.001, 1.006)*	1.010 (0.996, 1.024)	1.004 (0.997, 1.011)
	Lag 3	0.998 (0.996, 1.001)	0.994 (0.980, 1.007)	0.996 (0.990, 1.002)
	Lag 4	1.001 (0.999, 1.002)	0.994 (0.981, 1.007)	1.010 (1.004, 1.015)***
	Lag 01	1.003 (0.992, 1.013)	1.022 (0.982, 1.064)	0.994 (0.958, 1.031)
	Lag 02	0.987 (0.975, 0.998)*	0.970 (0.920, 1.024)	1.037 (0.990, 1.087)
	Lag 03	1.006 (0.993, 1.020)	0.964 (0.898, 1.036)	0.981 (0.926, 1.039)
	Lag 04	1.001 (0.992, 1.011)	1.049 (0.990, 1.112)	0.998 (0.961, 1.037)
After adjusting for collinearity	Lag 0	1.002 (0.997, 1.006)***	0.993 (0.977, 1.009)	0.995 (0.979, 1.011)
	Lag 1	1.002 (0.997, 1.006)	0.999 (0.983, 1.015)	0.989 (0.975, 1.003)
	Lag 2	1.003 (1.000, 1.006)*	1.009 (0.996, 1.023)	1.001 (0.993, 1.009)
	Lag 3	0.998 (0.996, 1.001)	0.994 (0.980, 1.007)	0.996 (0.990, 1.003)
	Lag 4	1.000 (0.998, 1.002)	0.992 (0.980, 1.004)	1.009 (1.003, 1.015)**
	Lag 01	1.004 (0.993, 1.014)	1.026 (0.986, 1.068)	0.990 (0.952, 1.029)
	Lag 02	0.983 (0.971, 0.995)**	0.972 (0.921, 1.025)	1.054 (1.004, 1.108)*
	Lag 03	1.009 (0.995, 1.024)	0.959 (0.892, 1.030)	0.974 (0.918, 1.034)
	Lag 04	1.001 (0.991, 1.011)	1.057 (0.998, 1.120)	0.998 (0.960, 1.039)

*** *P* < 0.001; **P < 0.01; *P < 0.05

### Subgroup statistical analysis results

To understand the detailed effects of air pollution on the city’s population, the data was stratified into gender and age (Table 5). In regards to gender, an increase in every 10 μg/m^3^ of PM_2.5_ and SO_2_ concentrations were significantly associated with risk of respiratory mortality in males. Only PM_2.5_ was significantly associated risk of respiratory mortality in females. No significant association was noted for exposure to O_3_ in the gender subgroups. In regards to age, a significant association was observed for exposure to PM_2.5_ in the ≤ 64 years subgroup, while no significant effect was noted for other pollutants. The ≥ 65 years subgroup showed no significant risk of respiratory mortality due to PM_2.5_, SO_2_, or O_3_ exposure.

**Table 5:** Gender and age-specific relative risk associated with 10 μg/m^3^ increase in air pollution concentration

Category	PM_2.5_	O_3_	SO_2_
Male	1.325 (0.990, 1.834)*	1.826 (0.435, 15.130)	1.555 (0.832, 3.868)*
Female	1.318 (0.894, 2.048)*	1.965 (0.336, 27.392)	1.365 (0.599, 5.636)
≤ 64 years	1.341 (0.802, 2.537)*	1.574 (0.143, 25.052)	1.495 (0.526, 7.938)
≥ 65 years	1.310 (0.890, 2.033)	1.424 (0.345, 24.444)	1.601 (0.694, 5.297)

*P < 0.05

### Subgroup lag effects

**Fig. 3:**
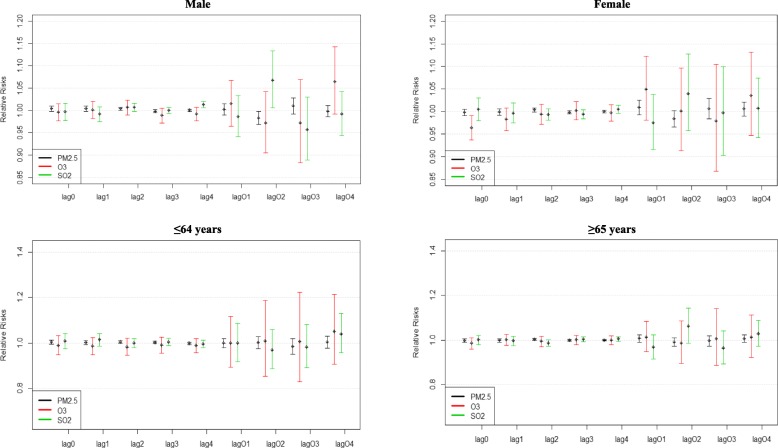
Gender and age lag-response relationship associated with 10 μg/m^3^ increase of ambient air pollutants

To reveal the delayed effects of air pollutants exposure on respiratory mortality in the specific subgroups, gender- and age-specific lag analysis were conducted. As shown in Fig. 3, each subgroup had a distinct lag pattern, with major differences observed between the single-day and cumulative lags. The single-day lags for all pollutants showed a more stable lag effect than the cumulative lags since their effects did not fluctuate greatly from their initial point. Although the single-day lags were seen to maintain a slightly stable lag effect, only lag 2 [1.004(1.000, 1.007), *P* = 0.0463] in males (PM_2.5_), lag 4 [1.012(1.005, 1.019), *P* = 0.00046] in males for (SO_2_) and lag 0 [0.964(0.938, 0.991), *P* = 0.0087] in females (O_3_) were statistically significant. No significant single-day lag effect was observed for the age subgroups. Cumulative lag effect was only noted to be statistically significant at lag 02 in males for PM_2.5_ [0.983(0.968, 0.998), *P =* 0.0246] and SO_2_ [1.068(1.006, 1.134), *P =* 0.0322]. No significant cumulative lag effect was noted for females and age subgroups.

## Discussion

In recent times, the formation of air pollution control guidelines and policy concerning health have been primarily based on epidemiological studies which reveal the adverse effects of air pollution on health. However, the struggle of pinpointing certain air pollutants to specific health outcomes remains. As it has been established, air pollution is a mixture of several pollutants, and these pollutants correlate either positively or negatively. The high correlation of air pollutants results in collinearity, making it difficult to determine the independent effect of a single pollutant. Most studies have faulted in properly adjusting for collinearity and thereby do not evaluate the accurate independent effect of each pollutant. In this study, keen attention was devoted to adjusting for collinearity using the principle component method. Results showed an increase in the effect estimates for all pollutants after adjusting for collinearity.

The current study aimed to explore a possible association between short term exposure to ambient air pollution and respiratory mortality in Xi’an. The results showed that daily ambient air pollutants had clear seasonal differences with PM_2.5_ and SO_2_ levels higher during the cold seasons. This seasonal trend validates the continued reliance on coal combustion for energy source particularly during winter, in addition to the increase of motor vehicles resulting in elevated traffic emissions in Xi’an [23, 24]. In contrast, O_3_ was higher during the warm seasons; this trend is valid given that photochemical reaction from which O_3_ is given off occurs more in the warm seasons due to the presence of sunlight (radiation). The hot and windy nature of the warm seasons also results in further suspension and dispersion of O_3_ [25]. The varying temporal trends between the studied ambient air pollutants may be due to different emission sources and possible confounding meteorological parameters such as temperature and relative humidity [26]. The role of temperature in aggravating mortality as well as the effects of air pollutants on health has been noted in some studies [27–29]. A study by Middleton et al. revealed that ozone was responsible for respiratory hospital admissions in Cyprus during the warm season [30]. Of the three assessed ambient air pollutants, only PM_2.5_ exceeded the annual average of the National Ambient Air Quality Standard (*GB3095–2012*) in China [22]. In this study, respiratory mortality was generally higher during winter and lower during summer.

Globally, respiratory diseases are on the rise, and various epidemiological studies report that short-term exposure to ambient air pollution causes adverse health effects, including an increase in respiratory mortality [5, 7, 31]. The three pollutants observed in this study showed positive associations with respiratory mortality. The effect estimates were consistently high before and after adjustment for collinearity by principal component analysis, with ozone having the highest estimates. However, only PM_2.5_ and SO_2_ associations with respiratory mortality were statistically significant (*P* < 0.05). From both associations, the effect estimates of SO_2_ was higher than PM_2.5_.

In comparison to studies carried out in other cities, our results had similar trends. A study done in the Pearl River Delta Region [32] associated exposure to PM_2.5_ and respiratory mortality with estimates of 1.68(1.00, 2.37), while ours was 1.313(1.032, 1.708). Another study in Wuhan [33] associated exposure to SO_2_ and respiratory mortality with estimates of 1.9(0.2, 3.6) covering a more extensive effect range than ours 1.402(0.827, 2.854). Although the association for O_3_ in our study was not statistically significant in the general analysis, the relative risk was however high in the single model and after adjusting for collinearity. Quite a few studies have also reported high effect estimates of O_3_ concerning respiratory mortality. A study in Shenyang [34] reported a high and statistically significant effect estimate of 4.7(0.00, 9.9). The non-significance of O_3_ may be attributed to several factors including concentration, duration of exposure and susceptibility of the population [35].

We investigated the lag relationship of ambient air pollution and respiratory mortality using a single day lag (0–4) and cumulative lag (01–04). For all observed pollutants, the effect estimates of the cumulative lag days were slightly larger than those of the single day lags, which suggests that accumulated exposure of the air pollutants increases the risk of respiratory mortality, a phenomenon that was noted in other studies as well [36]. Of all the pollutants, PM_2.5_ observed more statistically significant lags than other air pollutants. Its best lag noted was lag 2 [1.003(1.000, 1.006)], which was statistically significant and remained unchanged after adjusting for collinearity by principal component analysis; indicating that the confounding effect of other pollutants did not influence the lag effects of PM_2.5_. Other studies have also reported statistically significant single model lag effects (precisely lag 2) on respiratory mortality [19, 37–39]. SO_2_ observed its best lag at lag 4, with a slight decrease in lag effect estimate after the adjustment for collinearity. Although the relative risk for O_3_ multi-pollutant model increased when compared to the single pollutant model, no statistically significant lag effect was observed for ozone in the general lag analysis. Above all, sensitivity analysis of this study showed that effects due to lag exists, further proving that the temporal aspect of air pollution on health outcomes should be a critical factor in the analysis of environmental stressors and health.

Gender and age have been noted as effect modifiers in health assessments and have somewhat been standardized as proper means of population stratification. In this study, subgroup analysis revealed that every 10 μg/m^3^ increase of PM_2.5_ concentration was significantly associated with risk of respiratory mortality in the male, female and ≤ 64 years subgroups, no statistically significant association was noted for the ≥ 65 years subgroup. The male subgroup showed a slightly higher relative risk with a narrow confidence interval, as compared to the female’s which was lower with a wider confidence interval. This suggests the effect in the male subgroup was stronger and more precise than the female subgroup. SO_2_ was statistically associated with respiratory mortality in the male subgroup only. Similar trends of higher relative risk for males and ≤ 64 years and lower estimates for females and ≥ 65 years was also noted for SO_2_. Although some studies have reported stronger associations of these pollutants in females than males and some consider the ≥ 65 years subgroup more vulnerable than the ≤ 64 years, the differences in these results could be attributed to location, pollution concentration, population size as well as other underlying health factors associated with the population.

To observe the aspect of delayed effects within the population, the lag-response relationship for the subgroups was analyzed. The graphical presentation of the relationship (Fig. 2) showed that the effects of each pollutant in regard to each subgroup did not change or fluctuate greatly in the single day lags. The effects of the single day lag also maintained a narrow interval. In contrast, the cumulative lag showed changes in effects per lag and had a wider interval. The lag-response analysis also revealed a statistically significant association of O_3_ and females at lag 0 [0.964(0.938, 0.991)], which was the only significant association (*P* = 0.0087) noted for ozone in this study. Most studies have reported associations between ozone and females; a meta-analysis by Bell et al. [40] revealed that mortality due to ozone exposure was noted more in females [1.12(0.62, 1.63)] than males [0.73(0.40, 1.07)].

## Conclusion

Summarizing this report, short-term exposure to ambient fine particulate matter, ozone, and sulfur dioxide were significantly associated with respiratory mortality in Xi’an from 2014 to 2016. Assessing the effects of the pollutants in a single model as well as after adjusting for collinearity revealed significant associations between PM_2.5_ and SO_2_ and respiratory mortality. Furthermore, the observation of delayed effects via a lag-response analysis revealed a significant association between ozone and respiratory mortality only in females at lag 0. The adverse effects of air pollution noted in this study reveals the urgent need for more stringent control policies on the emission of air pollutants in Xi’an.

## Strengths and limitations

The main strength of our study was assessing more pollutants in relation to respiratory mortality and stratifying the data according to subgroups (gender and age). Respiratory mortality was diagnosed and classified based on ICD-10 codes which eliminated the possibility misclassification of cases and measurement errors; however, when interpreting the results, such possible errors should be taken into consideration. The limitation of the study was that pollution levels used were based on city-wide averages (reflecting background exposure levels) rather than personal exposure measurements. As a consequence, this was expected to result in exposure measurement error, and bias in terms of the risk estimates precision and strength. Ambient air pollutants (levels) were linked with respiratory mortality events and meteorological parameters based on admission dates and the root cause of disease, rather than the symptom on-set date and cause of mortality, which could cause a non-differential error in exposure measurement and bias of the effect estimates toward the null.

## Data Availability

The data-sets generated and or analyzed during the current study are not publicly available due to authors not having the right or permission to distribute the data due to its sensitive nature (i.e., patient-doctor confidentiality).

## Abbreviations

CI: Confidence interval
df: Degrees of freedom
ICD-10: International Classification of Diseases, Tenth Revision
NCD’s: Non-communicable diseases
O_3_: Ozone
PM_2.5_: Particulate matter (aerodynamic diameter less than 2.5 μm)
SO_2_: Sulfur dioxide
